# *MRE11* Function in Response to Topoisomerase Poisons Is Independent of its Function in Double-Strand Break Repair in *Saccharomyces cerevisiae*

**DOI:** 10.1371/journal.pone.0015387

**Published:** 2010-10-28

**Authors:** Nicolle K. Hamilton, Nancy Maizels

**Affiliations:** 1 Department of Immunology, University of Washington School of Medicine, Seattle, Washington, United States of America; 2 Department of Biochemistry, University of Washington School of Medicine, Seattle, Washington, United States of America; National Cancer Institute, United States of America

## Abstract

Camptothecin (CPT) and etoposide (ETP) trap topoisomerase-DNA covalent intermediates, resulting in formation of DNA damage that can be cytotoxic if unrepaired. CPT and ETP are prototypes for molecules widely used in chemotherapy of cancer, so defining the mechanisms for repair of damage induced by treatment with these compounds is of great interest. In *S. cerevisiae*, deficiency in *MRE11*, which encodes a highly conserved factor, greatly enhances sensitivity to treatment with CPT or ETP. This has been thought to reflect the importance of double-strand break (DSB) repair pathways in the response to these to agents. Here we report that an *S. cerevisiae* strain expressing the *mre11-H59A* allele, mutant at a conserved active site histidine, is sensitive to hydroxyurea and also to ionizing radiation, which induces DSBs, but not to CPT or ETP. We show that *TDP1*, which encodes a tyrosyl-DNA phosphodiesterase activity able to release both 5′- and 3′-covalent topoisomerase-DNA complexes *in vitro*, contributes to ETP-resistance but not CPT-resistance in the *mre11-H59A* background. We further show that CPT- and ETP-resistance mediated by *MRE11* is independent of *SAE2*, and thus independent of the coordinated functions of *MRE11* and *SAE2* in homology-directed repair and removal of Spo11 from DNA ends in meiosis. These results identify a function for *MRE11* in the response to topoisomerase poisons that is distinct from its functions in DSB repair or meiotic DNA processing. They also establish that cellular proficiency in repair of DSBs may not correlate with resistance to topoisomerase poisons, a finding with potential implications for stratification of tumors with specific DNA repair deficiencies for treatment with these compounds.

## Introduction

Topoisomerase poisons are potent drugs for treatment of cancer. Two naturally occurring topoisomerase inhibitors, camptothecin (CPT) and etoposide (ETP), are prototypes for this class of chemotherapeutics, which target Topoisomerase I (Topo I) and Topoisomerase II (Topo II) respectively. Topo I regulates DNA superhelicity ahead of transcription and replication forks by inserting itself into one strand of the DNA backbone, forming a 3′-covalent bond between a tyrosine residue on the enzyme and the nicked DNA strand and enabling rotation of the intact strand around the nick. CPT binds at the single-strand break 3′ of the Topo I-DNA complex, stabilizing the covalent Topo I-DNA intermediate and inhibiting religation of the DNA nick. Topo II promotes decatenation of DNA following replication, inserting into both strands of the duplex to form a double-strand break (DSB), with 5′-DNA ends tethered to tyrosine residues on Topo II subunits, enabling intact DNA to pass through the DSB. ETP intercalates at Topo II insertion sites, stabilizing the DSB intermediate. Defining the mechanisms of repair of damage induced by CPT and ETP is of considerable practical importance, because both are potent cytotoxic agents and their derivatives are commonly used in cancer chemotherapy.

Mutants of *S. cerevisiae* deficient in *MRE11* (*mre11Δ*) are very sensitive to CPT [1], [2], [3] and to ETP [4]. *MRE11* encodes a multifunctional nuclease, active as a 3′-5′ dsDNA exonuclease, a single-strand DNA endonuclease and an AP lyase *in vitro*, and shown to function in DNA repair, meiotic recombination, telomere maintenance and immunoglobulin gene diversification [5], [6], [7], [8], [9], [10], [11]. Its mechanism of function in response to CPT or ETP is not understood, but has been thought to correlate with activity in DSB repair, which is dependent upon its 3′-5′ exonuclease activity.

Here we describe a new *S. cerevisiae* mutant allele, *mre11-H59A*. We show that an *S. cerevisiae mre11-H59A* strain is as sensitive to HU and IR as the well-characterized DSB repair-deficient *mre11-H125N* strain [3], [10], [12], [13]. However, in contrast to the *mre11-H125N* strain, the *mre11-H59A* strain is resistant to CPT. CPT-resistance does not depend upon *TDP1*, which encodes a factor that releases covalent 3′- or 5′-tyrosyl DNA bonds *in vitro* [14], [15], [16], [17]. The *mre11-H59A* and *mre11-H125N* strains are both resistant to ETP, which is toxic to the *mre11Δ* strain; but deficiency in *TDP1* (*tdp1Δ*) causes these strains to become ETP-sensitive. Neither CPT- nor ETP-resistance of the *mre11-H59A* strain depends upon *SAE2*, which is required for *MRE11*-dependent removal of Spo11 from the ends of meiotic DNA [18] and regulates functions of *MRE11* in homologous recombination [19]. Thus *MRE11* has distinct functions in repair of damage induced by topoisomerase poisons and repair of mitotic and meiotic DSBs.

## Results

### *S. cerevisiae mre11-H59A* Is Sensitive to Hydroxyurea but Not CPT

**Figure 1:**
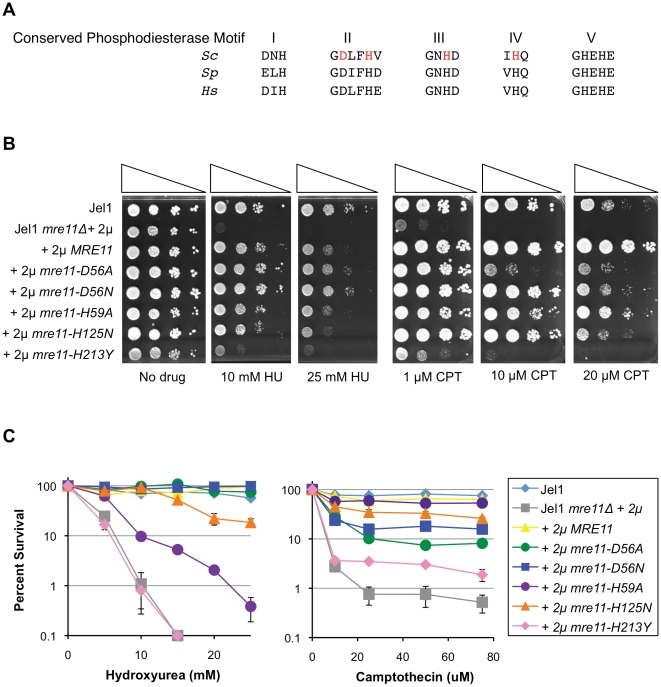
The *mre11-H59A* allele confers sensitivity to HU but not CPT. A. Conserved phosphodiesterase motifs in the active site of the Mre11 protein. Sequences of conserved amino acid residues in Motifs I–V are shown for three different organisms: *S. cerevisiae* (*Sc*), *S. pombe* (*Sp*), and *Homo sapiens* (*Hs*). Effects of mutations at *S. cerevisiae* residues shown in red in were tested in experiments described herein. The lengths of the Mre11 polypeptides are all different, and positions of the conserved residues in each motif are shown below for reference: *Sc*, I, H18; II, D56, H59; III, H125; IV, H213; V, H241, H243; *Sp*, I, H13; II, D65, H68; III, H134; IV, H222; V, H250, H252; *Hs*, I, H22; II, D60, H63; III, H129; IV, H217; V, H245, H247. B. Serial spot dilution assays of sensitivity to HU and CPT of *S. cerevisiae* wild-type parental line Jel1 (Jel1); or its *MRE11*-deficient derivative, Jel1 *mre11Δ*, stably transformed with 2 µ plasmid vectors expressing protein as indicated, including empty vector (Jel1 *mre11Δ* +2 µ), wild-type *MRE11* (+2 µ *MRE11*), or indicated mutant alleles (+2 µ *mre11-D56A*, +2 µ *mre11-D56N*, +2 µ *mre11-H59A*, +2 µ *mre11-H125N*, +2 µ *mre11-H213Y*). Cells were spotted at 10-fold serial dilutions (indicated by triangles) on rich plates containing no drug (left) or indicated concentrations of HU or CPT. C. Clonogenic survival assays of sensitivity to HU and CPT. Strains were assayed for colony formation at indicated doses of each compound. Survival was normalized to untreated samples; error bars indicate standard error of the mean.

To distinguish functions of *MRE11* in DSB repair and response to DNA damage by topoisomerase poisons, two previous findings drew our attention to the conserved active site histidine at residue 59 (H59) in phosphodiesterase motif II of *S. cerevisiae* Mre11 (Fig. 1A). Biochemical analysis had shown that purified recombinant *Pyrococcus furiosus* Mre11 with a mutation at this site (H52S) is deficient in exonuclease activity; while genetic analysis had shown that mutation at the corresponding position of the *Schizosaccharomyces pombe* Mre11 homologue (*rad32-H68S*) did not render cells sensitive to CPT, HU or IR [8].

In order to study the effect of mutation of H59 in *S. cerevisiae* and compare this allele to well-characterized mutant alleles, we generated a panel of strains bearing mutations in conserved residues in the Mre11 active site phosphodiesterase motifs (Fig. 1A). *S. cerevisiae* Jel1 *mre11Δ* (LSY1706, *MATα leu2 trp1 ura3-52 prb1-1122 pep4-3 his3::GAL10-GAL4 mre11::His3MX6*; [12]) was stably transformed with pYES 2 µ vectors expressing C-terminal TAP-tagged Mre11 or mutant Mre11-H59A, Mre11-D56A, Mre11-D56N, Mre11-H125N or Mre11-H213Y under control of the *GAL1* promoter, and gene and protein expression were confirmed by RT-PCR (not shown) and western blotting (Fig. S1). Mutations at D56 alter a conserved glutamate in close proximity to H59 in phosphodiesterase motif II (Fig. 1A), known to be important for resistance to HU and IR [12]. Mutation of H125 alters a residue in phosphodiesterase motif III that is invariant among species and necessary for stabilizing the transition state during nucleolysis [8], [20], [21]. *S. cerevisiae mre11-H125N* mutants have previously been shown to be sensitive to DNA damaging agents including CPT and IR [3], [10], [12], [13]; and a corresponding *S. pombe* active site mutant (*rad32-H134S*) is associated with both CPT-sensitivity and IR-sensitivity [8]. The *mre11-H213Y* allele, one of the first *MRE11* mutants studied, alters an invariant histidine in motif IV. It causes deficiency in both meiotic and mitotic DSB repair [22], and the corresponding mutant Mre11 protein fails to form a stable complex with Rad50 [12], [23], [24].

Sensitivity to HU and CPT was determined by spot dilution assays. Serial 10-fold dilutions of an overnight culture of each strain were spotted onto YPD plates containing various concentrations of HU or CPT. The Jel1 *mre11Δ* strain and its derivative expressing *mre11-H213Y* were extremely sensitive to HU; the derivatives expressing *mre11-H59A* and *mre11-H125N* were no more sensitive than the derivative expressing *MRE11*; and the derivatives expressing *mre11-D56A* and *mre11-D56N* were relatively resistant (Fig. 1B, left). Spot tests also showed that Jel1 *mre11Δ* and its derivative expressing *mre11-H213Y* were extremely sensitive to CPT, the derivatives expressing *mre11-D56A*, *mre11-D56N* and *mre11-H125N* slightly less sensitive, and the derivative expressing *mre11-H59A* relatively resistant, although slightly less so than the derivative expressing *MRE11* (Fig. 1B, right). Identical results were obtained using the low copy number CEN plasmid p416ADH expressing *MRE11*, *mre11-H59A* or *mre11-H213Y* alleles (data not shown). Thus, the *mre11-H59A* mutation caused sensitivity to HU but not to CPT, as measured by spot dilution assays.

The contrasting effects of the *mre11-H59A* mutation on HU and CPT sensitivity were confirmed by clonogenic survival assays. These assays showed that the strain expressing *mre11-H59A* was sensitive to HU, less so than the extremely HU-sensitive Jel1 *mre11Δ* strain or its derivative expressing *mre11-H213Y*, but more sensitive than the parental Jel1 strain or the Jel1 *mre11Δ* derivatives expressing *MRE11*, *mre11-D56A*, *mre11-D56N* or *mre11-H125N* (Fig. 1C, left). Nonetheless, the Jel1 *mre11Δ* derivative expressing *mre11-H59A* was as resistant to CPT as the Jel1 parental line or the Jel1 *mre11Δ* derivative expressing *MRE11* (Fig. 1C, right).

### The *S. cerevisiae mre11-H59A* Strain Exhibits Intermediate Sensitivity to IR

**Figure 2:**
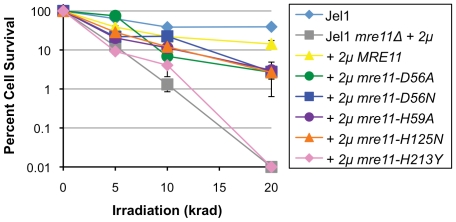
The *mre11-H59A* allele confers sensitivity to IR. Cell survival was measured following indicated exposure to radioactive ^137^Cs, and normalized to unirradiated samples. Error bars indicate standard error of the mean. Strain notations as in Fig. 1.

To test sensitivity to IR, we focused on a panel of six strains, exposed cells in liquid culture to radioactive cesium, plated aliquots in triplicate, determined colony number after 3–4 days growth and normalized to the unexposed samples. The *mre11-H59A* strain was of intermediate sensitivity to IR, less resistant than the Jel1 parental strain or the Jel1 *mre11Δ* derivative expressing *MRE11*, but comparable to the derivatives expressing *mre11-D56N*, *mre11-D56A* or *mre11-H125N*, and more resistant than Jel1 *mre11Δ* or its derivative expressing *mre11-H213Y* (Fig. 2).

### *TDP1* Does Not Contribute to CPT-Resistance of *MRE11*-Deficient Strains

**Figure 3:**
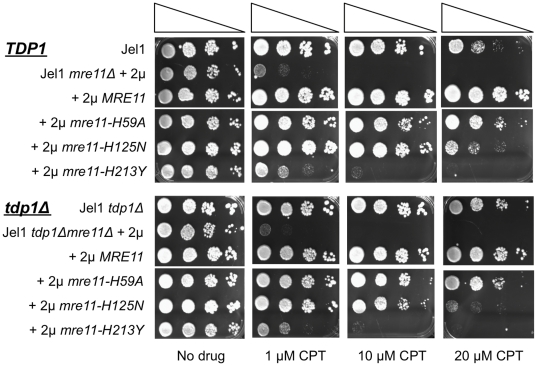
*TDP1* does not contribute to CPT-resistance of *MRE11*-deficient strains. Serial spot dilution assays of sensitivity to CPT of *S. cerevisiae* Jel1 *TDP1* or Jel1 *TDP1 mre11Δ* (above), and Jel1 *tdp1Δ* or Jel1 *tdp1Δ mre11Δ* (below). The *mre11Δ* derivatives were stably transformed with 2 µ plasmid vectors, including empty vector (Jel1 *mre11Δ* +2 µ) or vectors expressing wild-type *MRE11* (Jel1 *mre11Δ* +2 µ *MRE11*) or indicated mutant alleles (+2 µ *mre11-H59A*, +2 µ *mre11-H125N*, +2 µ *mre11-H213Y*). Cells were spotted at 10-fold serial dilutions on rich plates containing no drug (left) or indicated concentrations of CPT.

The enzyme Tyrosyl-DNA-phosphodiesterase 1 (Tdp1) was identified as an activity in *S. cerevisiae* extracts capable of removing a trapped Topo I-DNA complex *in vitro* [14]. However, *S. cerevisiae tdp1Δ* mutants exhibit only minor increases in sensitivity to CPT, either in strains expressing *MRE11* or in the *MRE11*-deficient strains tested thus far, including *mre11-H125N* [1], [3], [17], [25]. To compare function of *TDP1* and *MRE11* in CPT resistance, we created Jel1 *tdp1Δ* and Jel1 *mre11Δ tdp1Δ* derivatives, and transformed the latter with 2 µ plasmids expressing *MRE11* or *mre11-H59A, mre11-H125N* and *mre11-H213Y* mutant alleles. In spot dilution assays, the Jel1 *mre11Δ* strain was much more severely CPT-sensitive than the Jel1 *tdp1Δ* strain, and sensitivity was not enhanced in the Jel1 *mre11Δ tdp1Δ* double mutant (Fig. 3). Moreover, the Jel1 *tdp1Δ mre11Δ* derivatives expressing *MRE11, mre11-H59A, mre11-H125N* or *mre11-H213Y* exhibited CPT-sensitivity essentially indistinguishable from the corresponding Jel1 *mre11Δ* derivatives (Fig. 3, compare lower and upper). Thus, *MRE11* was more critical to CPT-resistance than *TDP1*, and deficiency in *TDP1* did not affect CPT sensitivity of *MRE11*-deficient strains.

### *TDP1* Contributes to ETP-Resistance of *mre11-H59A* and *mre11-H125N* Mutants

**Figure 4:**
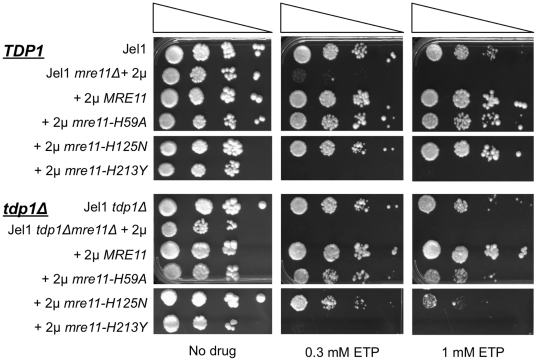
*TDP1* contributes to ETP-resistance of *mre11-H59A* and *mre11-H125N* strains. Serial spot dilution assays of sensitivity to ETP of indicated *S. cerevisiae* derivatives of parental line Jel1; notations as in Fig. 3. Cells were spotted at 10-fold serial dilutions on rich plates containing no drug (left) or indicated concentrations of ETP.

In *S. cerevisiae*, *MRE11*-deficiency causes sensitivity to ETP, which traps covalent complexes formed by Topo II with DNA 5′-ends [4]. We therefore tested ETP sensitivity of a panel of *MRE11*-deficient strains in a spot dilution assay. We found that the Jel1 *mre11Δ* strain and its derivative expressing *mre11-H213Y* were extremely sensitive to ETP, while derivatives expressing *mre11-H59A* or *mre11-H125N* were as resistant as Jel1 or the Jel1 *mre11Δ* derivative expressing *MRE11* (Fig. 4, above). Thus, expression of *mre11-H59A* conferred resistant to ETP and CPT, despite marked sensitivity to HU and IR; and expression of *mre11-H125N* strain conferred resistant to ETP, despite sensitivity to CPT, HU and IR.

Tdp1 can remove not only Topo I-3′-DNA covalent complexes *in vitro*, but also Topo II-5′-DNA covalent complexes [17]. We therefore asked how ETP-resistance was affected by deficiencies in *TDP1* and *MRE11*. In spot dilution assays, Jel1 and Jel1 *tdp1Δ* were comparably ETP-sensitive, while the Jel1 *mre11Δ* strain was much more severely ETP-sensitive than the Jel1 *tdp1Δ* strain (Fig. 4, compare lower and upper). Thus, deficiency in *MRE11* caused much more severe ETP-sensitivity than deficiency in *TDP1*. Moreover, while deficiency in *TDP1* did not affect ETP-sensitivity of the Jel1 *mre11Δ* derivative expressing *MRE11*, it did cause a modest increase in ETP-sensitivity of the Jel1 *mre11Δ* derivative expressing *mre11-H59A*, and a more severe increase in sensitivity of the derivative expressing *mre11-H125N* (Fig. 4, lower).

### *SAE2* Makes a Modest Contribution to CPT- or ETP-Resistance

**Figure 5:**
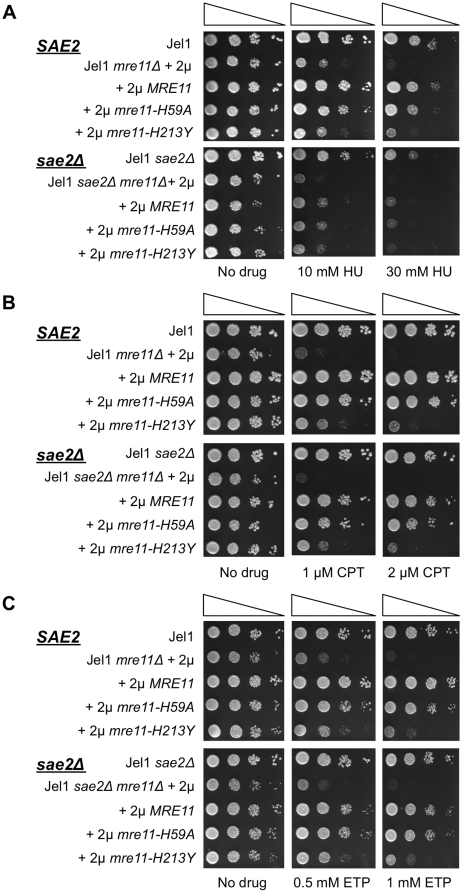
*SAE2* contributes to HU-resistance, but not CPT- or ETP-resistance. A. Serial spot dilution assay of HU sensitivity of *S. cerevisiae* parental line Jel1; or its *mre11Δ* and *sae2Δmre11Δ* derivatives stably transformed with 2 µ plasmid vectors, including empty vector (Jel1 *mre11Δ* +2 µ) or vectors expressing wild-type *MRE11* (Jel1 *mre11Δ* +2 µ *MRE11*) or indicated mutant alleles (+2 µ *mre11-H59A*, +2 µ *mre11-H213Y*). B. Serial spot dilution assay of CPT sensitivity of the strains described in panel A. C. Serial spot dilution assay of ETP sensitivity of the strains described in panel A.

*SAE2* encodes a repair endonuclease that is essential for Mre11 function in removal of Spo11 from 5′-ends of DNA in meiosis, and that regulates activities of Mre11 in homologous recombination [18], [19], [26]. Deficiency in *SAE2* has been reported not to affect CPT- or ETP-sensitivity in *S. cerevisiae* [3], [18], although the *SAE2* ortholog, *CtIP*, is essential for CPT-resistance in *S. pombe* [27]. Because of the role of *SAE2* in meiosis, it seemed important to test the effect of deficiency in *SAE2* on CPT- and ETP-sensitivity of the Jel1 *mre11Δ* derivative expressing *mre11-H59A*. We therefore generated Jel1 *sae2Δ* and Jel1 *sae2Δ mre11Δ* strains, and Jel1 *sae2Δ mre11Δ* derivatives expressing *MRE11*, *mre11-H59A* or *mre11-H213Y* alleles (the latter was included as a control due to its severe meiotic defect). Consistent with the role of *SAE2* in DSB repair, strains deficient in *SAE2*, including Jel1 *sae2Δ*, Jel1 *sae2Δ mre11Δ*, and derivatives of the latter expressing *MRE11, mre11-H59A, or mre11-H213Y*, all exhibited greater HU sensitivity than the parental *SAE2* strains (Fig. 5A). However, deficiency in *SAE2* caused only a very modest increase in CPT-sensitivity (Fig. 5B) or ETP-sensitivity (Fig. 5C). Thus, *SAE2* is not critical to *MRE11*-dependent repair of damage induced by CPT or ETP.

## Discussion

We have shown that the *S. cerevisiae mre11-H59A* strain is resistant to topoiosomerase poisons CPT and ETP, which create protein-DNA covalent complexes; but sensitive to HU and sensitive to IR, which induces DSBs. These results establish that the *MRE11* function in DSB repair can be separated from its function in repair of CPT- or ETP-induced DNA damage. They also establish that a cell need not be proficient in repair of DSBs to resist topoisomerase poisons.

Our results identify several clear distinctions between *S. cerevisiae* and *S. pombe* in the response to topoisomerase poisons. One difference is in functions associated with specific residues of the highly conserved active site of the Mre11 polypeptide. The *S. cerevisiae mre11-H59A* strain that we have studied carries a mutation at a conserved active site histidine in phosphodiesterase motif II of the active site and is resistant to CPT but sensitive to HU and IR. An *S. pombe* strain carrying a mutation at the corresponding position, *rad32(mre11)-H68S*, is resistant to CPT and also to HU and IR [8].

The repair pathways for CPT- and ETP-induced damage in *S. cerevisiae* and *S. pombe* also exhibit distinct dependence upon other factors. In *S. cerevisiae*, *TDP1*-deficiency has little effect on CPT-sensitivity, either in strains expressing wild-type *MRE11* [1], [25] or in the four *mre11* mutant strains we examined. This contrasts with *S. pombe*, where *TDP1*-deficiency has a pronounced effect on CPT-sensitivity [28]. In *S. cerevisiae*, *SAE2* makes only a very modest contribution to CPT- or ETP-resistance. This also contrasts with *S. pombe*, where deficiency in the *SAE2* ortholog, *CtIP*, impairs the ETP response, but surprisingly promotes the CPT response [27].

How might *MRE11* function to prevent toxicity by topoisomerase poisons independent of DSB repair? A critical step that distinguishes the response to topoisomerase poisons from other cytotoxic treatments is removal of the covalent protein-DNA complexes that accumulate in cells treated with these compounds. Our results raise the possibility that *MRE11* may promote cleavage of tyrosyl-DNA bonds, either directly or indirectly. Several factors possess this activity, including the conserved enzyme Tdp1, which can cleave both 3′- and 5′-covalent tyrosyl-DNA bonds *in vitro* [14], [15], [16], [17]; the structure-specific nucleases Rad1/Rad10 and Mms4/Mus81, which appear to function redundantly with Tdp1 in release of 3′-covalent protein DNA complexes *in vivo* [29]; and the recently discovered human factor, Tdp2, which can cleave 5′-tyrosyl-DNA covalent bonds *in vitro* and rescue CPT-sensitivity of *S. cerevisiae tdp1Δ rad1Δ* mutants *in vivo* [30]. Consistent with a role for *MRE11* in promoting cleavage of tyrosyl-DNA covalent bonds, topoisomerase-DNA complexes have been shown to persist in an *S. pombe rad32(mre11)-D65N* strain following treatment with CPT or ETP [27].

Determining the mechanism of *MRE11* function in the response to topoisomerase poisons has important implications for the clinical setting. A subset of human colorectal cancers is *MRE11*-deficient [31], [32]. If *MRE11* functions to promote release of covalent topoisomerase-DNA complexes independent of its role in DSB repair, *MRE11*-deficient tumors may be priority candidates for treatment with CPT and ETP derivatives. This could have implications for stratification of tumors with specific DNA repair deficiencies for treatment with topoisomerase poisons.

## Materials and Methods

### *S. cerevisiae* Strains

Strains were derived from Jel1 (*Matα leu2 trp1 ura3-52 prbl-1122 pep4-3 his3::pGAL10-GAL4*) or Jel1 *mre11Δ* (*Matα leu2 trp1 ura3-52 prbl-1122 pep4-3 his3::pGAL10-GAL4 mre11::HIS3*), kindly provided by Dr. Lorraine Symington, Columbia University [12]. The *tdp1Δ* and *sae2Δ* derivatives were constructed using the PCR method of gene deletion [33], which replaces the target gene by a G418 resistance marker. G418-resistance markers were amplified from *S. cerevisiae tdp1Δ* and *sae2Δ* strains, kindly provided by Dr. Stanley Fields (University of Washington). Primers, designed to provide homology arms of about 500 bp, were:

*SAE2* F: 5′-CAGTAATTGACGATGCGGAAGG

*SAE2* R: 5′-CGACGTTCTCTATCATAATAAAACCCTGG

*TDP1* F: 5′-CAGCATTTTTATGTTCAGTAATCATTGAACTTG

*TDP1* R: 5′-GGAGCATCTATTAAAAAGAGCTTTTAATC

PCR products were purified and transformed into Jel1 or *mre11Δ* strains using the lithium acetate method. Integrants were selected on either CSM-URA or YPD plates containing 200 µg/ml G418, and deletion verified by sequencing the products of colony PCR.

### *MRE11* Expression Constructs

*MRE11* was PCR-amplified from Jel1, using PCR primers 5′-CGGGGTACCATGGTGCATCATCAC and 5′-CTAGTCTAGATTTTCTTTTCTTAGCAAGGAGACTTCCAAGAATATCCG, which modified the gene to create a KpnI site and consensus translation initiation sequence at the 5′ end and remove the stop codon and create an XbaI site at the 3′ end (KpnI and XbaI sites underlined). DNA was digested with KpnI and XbaI and inserted into the KpnI and XbaI sites of a 2 µ pYES2 plasmid carrying the URA3 selectable marker (Invitrogen, Carlsbad, CA). A TAP tag was amplified from a construct provided by Dr. Trisha Davis (University of Washington), using primers 5′-CTAGTCTAGAATGAAGCGACGATGGAAAAAGAATTTCATAGCCG and 5′-CTAGTCTAGATTATTCTTTGTTGAATTTGTTATCCGCTTTCGGTGCTTGAG to create XbaI sites (underlined) on both 5′ and 3′ ends, XbaI-digested and inserted into the XbaI-digested pYES2-*MRE11* construct, and screened for directional insertion, generating pYES2-*MRE11-TAP*. *MRE11* was expressed from the GAL1 promoter in these plasmids. Mutants were generated by QuikChange (Stratagene, La Jolla, CA) and verified by sequencing, using the following primers and their complements (mutations underlined):

D56A: 5′-ATGGTTGTACAGTCCGGTGCTCTTTTTCACGTGAATAAG

D56N: 5′-ACATGGTTGTACAGTCCGGTAATCTTTTTCACGTGAATAAG

H59A: 5′-GTTGTACAGTCCGGTGATCTTTTTGCCGTGAATAAGCCTTC

H125N: 5′-TATTCGGCATATCAGGTAATAATGATGATGCGTCGGG

H213Y: 5′-GGTTTAATTTAATGTGCGTCTATCAAAATCATACAGGTCACAC

### Western Blotting

Approximately 5×10^8^ cells were pelleted, resuspended in 500 µl lysis buffer (25 mM Tris-HCl pH 7.5, 1 mM EDTA, 0.5% NP-40, 10% glycerol, 1 mM PMSF, 1 mM DTT, 150 mM NaCl, 1xRoche Complete Protease Inhibitor), then lysed by vortexing with 500 µl glass beads at full speed 5×1 min, incubating at least 1 min on ice between vortexing. Following SDS-PAGE electrophoresis and transfer, blots were probed using polyclonal anti-ScMre11 (GeneTex) at 1∶2000 and anti-β-actin (Abcam) at 1∶2000.

### HU, CPT, ETP and IR Sensitivity Assays

Published procedures were used to assay sensitivity to HU [12], CPT [1], IR [12], and ETP [4]. In brief, spot dilution assays were performed by plating 3 µl of serial 10-fold dilutions of a liquid culture, containing from 10^3^–10^6^ cells/ml, on YPD plates with or without drug. Clonogenic survival assays for CPT-sensitivity were carried out on cells cultured in liquid medium containing 1–20 µM CPT for 6 hr (about 5–8 cell divisions). Aliquots were removed and plated on YPD in triplicate, colonies counted 3–4 days later and percent survival normalized to an untreated control. For HU clonogenic survival assays, cells from liquid culture were plated in triplicate on YPD plates containing various concentrations of HU, colonies counted 3–4 days later and percent survival normalized to an untreated control. Sensitivity to IR was measured by exposing cells in liquid culture to radioactive ^137^Cs at the indicated dosage, plating on YPD and counting surviving colonies 3–4 days later. A JL-Shepherd Model 81-14 Cesium-137 Irradiator was used for all *in vitro* radiation treatments in this study. Cell suspensions in 1.5 ml tubes were exposed to ^137^Cs γ-rays at a dose rate of 3 Gy/min. Dosimetry was performed before every radiation treatment to ensure consistency in the dose rate. For survival curves, representative plots are shown for at least three independent experiments with similar results; error is calculated for triplicates within the experiment; and survival was normalized to untreated controls.

## Supporting Information Legends

Figure S1 **Expression of Mre11 protein.** Western blot analysis of Mre11 protein levels in indicated strains (designated as described in Fig. 1), normalized to β-actin. Mre11 expressed from 2 µ vectors carried a C-terminal TAP-tag, and was therefore slightly larger than the endogenous protein.
(TIF)
