# Supplementary figures and images for: MRE11 Function in Response to Topoisomerase Poisons Is Independent of its Function in Double-Strand Break Repair in Saccharomyces cerevisiae
Source: PLoS One. 2010 Oct 28;5(10):e15387. doi: 10.1371/journal.pone.0015387 (PMC2965672; doi:10.1371/journal.pone.0015387)

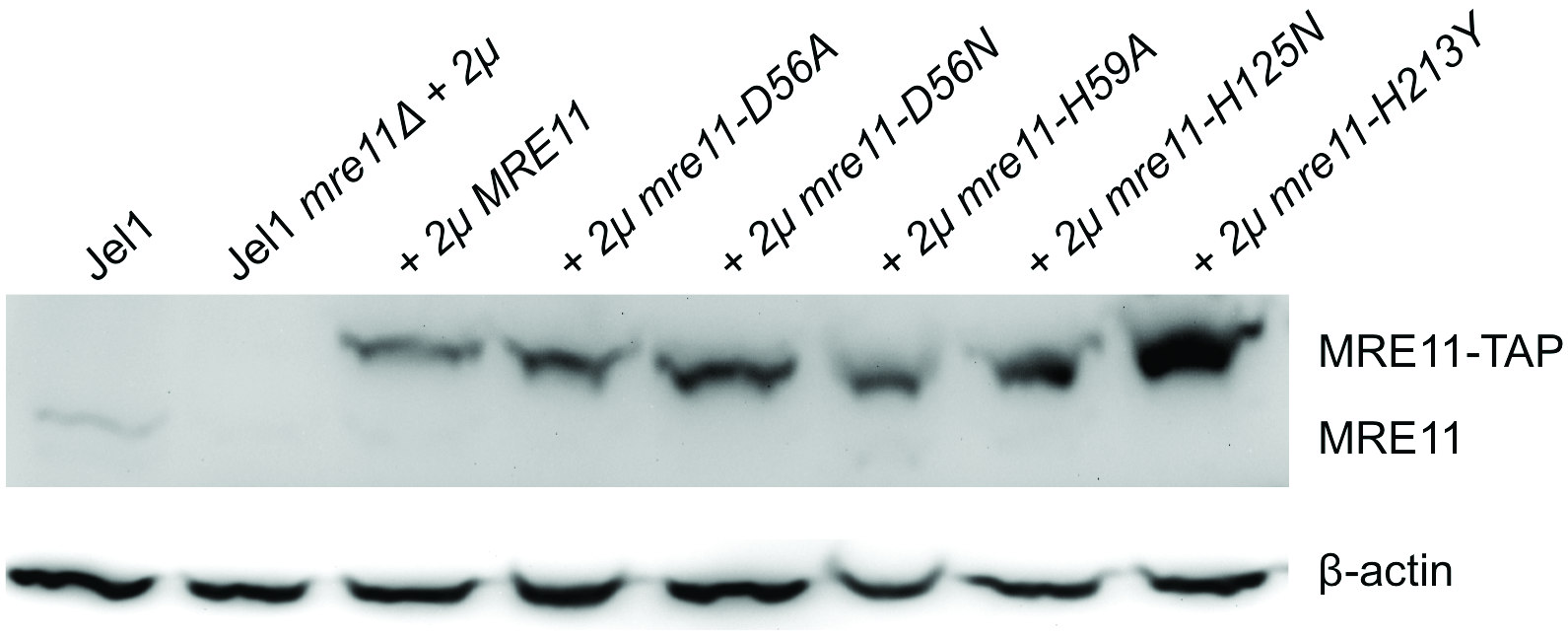

Supplement: Figure S1 — Expression of Mre11 protein. Western blot analysis of Mre11 protein levels in indicated strains (designated as described in Fig. 1), normalized to β-actin. Mre11 expressed from 2 µ vectors carried a C-terminal TAP-tag, and was therefore slightly larger than the endogenous protein. (TIF) [file pone.0015387.s001.tif]
